# MiR858b Inhibits Proanthocyanidin Accumulation by the Repression of *DkMYB19* and *DkMYB20* in Persimmon

**DOI:** 10.3389/fpls.2020.576378

**Published:** 2020-12-21

**Authors:** Sichao Yang, Meng Zhang, Liqing Xu, Zhengrong Luo, Qinglin Zhang

**Affiliations:** Key Laboratory of Horticultural Plant Biology, Ministry of Education, Huazhong Agricultural University, Wuhan, China

**Keywords:** persimmon, proanthocyanidin, microRNA, MYB, transformation

## Abstract

Persimmon proanthocyanidin (PA) biosynthesis is controlled by structural genes and regulated by transcription factors (TFs). MicroRNAs are a key factor involved in regulating gene expression at the posttranscriptional level whose functions in persimmon PA biosynthesis are poorly understood. Here, we identified a microRNA, miR858b, that putatively targets two R2R3-MYB TFs, *DkMYB19* and *DkMYB20*. *DkMYB19*, *DkMYB20*, and miR858b showed divergent expression patterns during fruit development, and the interaction between miR858b and *DkMYB19* or *DkMYB20* was experimentally validated by 5′ RNA ligase-mediated RACE, LUC enzyme activity analysis, and GFP signal detection. The DkMYB19 localized to the nucleus as well as the cytoplasm and DkMYB20 localized to the nucleus. The overexpression of miR858b led to the downregulation of *DkMYB19* and *DkMYB20*, which reduced the content of PA, whereas a reduction in miR858b activity upregulated *DkMYB19* and *DkMYB20*, resulting in a high content of PA in leaves transiently expressing a small tandem target mimic construct for blocking miR858 (STTM858b) *in vivo*. The transient transformation of miR858b in fruit discs *in vitro* also reduced the content of PA, while the content of PA increased under the transient transformation of fruit discs with STTM858b, *DkMYB19*, or *DkMYB20*. A similar phenomenon was observed upon the overexpression of miR858b in wild-type (WT) *Arabidopsis* and *DkMYB19* or *DkMYB20* in persimmon leaf calli. These findings suggested that miR858b repressed the expression of *DkMYB19* and *DkMYB20*, which contributed to the PA accumulation in persimmon.

## Introduction

Persimmon (*Diospyros kaki*), one of the major fruit crops in Asia, accumulates large amounts of PA in its fruit “tannin cells”,’ which causes a strong astringency sensation when fresh fruits are consumed. Persimmon varieties are classified into two groups according to the genetic characteristics of natural deastringency: pollination-constant non-astringent (PCNA) persimmon is a mutant phenotype that loses its astringency naturally during fruit development on the tree so that the fruits are edible when ripe, whereas the non-PCNA type requires artificial treatment to remove astringency, which involves carbon dioxide gas or ethanol vapor treatment or drying after peeling before consumption. The PCNA trait is recessive to the non-PCNA trait and is qualitatively inherited, which is controlled by a single gene known as the *AST*/*ast* allele (Akagi et al., 2009, 2011). However, the cultivated persimmon cultivars are hexaploidy and make the *AST*/*ast* allele segregation complicated in progeny.

The biosynthesis of proanthocyanidin (PA), also known as condensed tannin, involves both structural genes and transcription factors (TFs). These structural pathway genes encode anthocyanidin reductase (*ANR*) and leucoanthocyanidin reductase (*LAR*) (Akagi et al., 2009, 2011, 2012), multidrug and toxic compound extrusion (MATE) transporter (Zhao and Dixon, 2009), a glutathione S-transferase (GST) (*TT19*) (Kitamura et al., 2004), a proton pump (*auto-inhibited* H+-*ATPase isoform*) (*AHA10*) (Baxter et al., 2005), and laccase-like polyphenol oxidase (*TT10*) (Zhao et al., 2010). In persimmon, two MYB TFs, *DkMYB2* and *DkMYB4*, are involved in regulating PA in persimmon by activating the transcription of *DkF3′5′H* (flavonoid 3′5′ hydroxylase) and *DkANR* or *DkANR* and *DkLAR*, respectively (Akagi et al., 2009, 2010). *DkMYC1* may be an important bHLH TF that regulates PA biosynthesis (Su et al., 2012; Naval et al., 2016; Nishiyama et al., 2018), while *DkbZIP5* could directly regulate *DkMYB4* in an ABA-dependent manner by recognizing ABA-responsive elements in the promoter region of *DkMYB4* (Akagi et al., 2012). It follows that DkMYB2 or DkMYB4 could interact with DkMYC1 and DkWDR1 to form ternary MBW complexes that are involved in regulating PA accumulation in fruit (Naval et al., 2016).

A search of the literature suggests that the majority of plant miRNAs target TFs to play critical regulatory roles in multiple biological processes, such as development, primary and secondary metabolism, and stress responses (Wu, 2013; Sharma et al., 2016; Wang et al., 2016). For example, miR858 is a conserved plant miRNA that is involved in flavonoid metabolism in several plant species; the miR858a-MYB network is involved in flavonoid biosynthesis, plant growth, and development (Sharma et al., 2016), and the HY5-miR858a-MYBL2 loop is a cellular mechanism that modulates anthocyanin biosynthesis (Wang et al., 2016). In addition, the blockage of miR858 induces anthocyanin accumulation by modulating *SlMYB48-like* transcripts in tomato (Jia et al., 2015).

Proanthocyanidin is recognized as an important secondary metabolite in persimmon fruit during developmental stages, and the function of miRNA in PA biosynthesis is poorly understood. Here, we screened miR858b from the miRNA database of persimmon, which was predicted to target two MYB genes, *DkMYB19* and *DkMYB20*. The molecular dynamics of the expression of miR858b, *DkMYB19*, and *DkMYB20* were assessed together with the changes in PA content in Chinese PCNA (C-PCNA) persimmon. Our results suggested that miR858b and its target genes *DkMYB19* and *DkMYB20* show divergent expression patterns in persimmon fruit during development. In addition, our study provided evidence that miR858b-MYB plays an important role in the regulation of PA biosynthesis in persimmon.

## Materials and Methods

### Plant Materials

**FIGURE 1:**
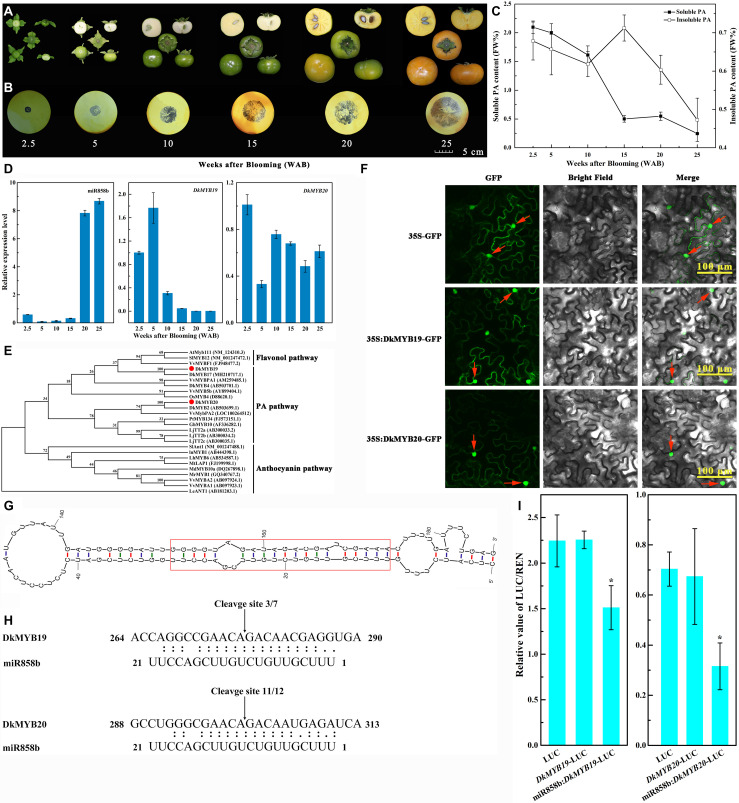
Identification and characterization of miR858b in ‘Eshi 1’ persimmon. **(A)** Representative photos showing fruits sampled at six stages: 2.5, 5, 10, 15, 20, and 25 WAB. **(B)** Analysis of the soluble proanthocyanidin (PA) content in the persimmon fruits via the imprinting method. **(C)** Measurement of the content of soluble and insoluble PA in the fruits by the Folin–Ciocalteau method. Errors bars indicate SEs from three biological replicates (*n* = 3). **(D)** Expression analysis of miR858b, *DkMYB19*, and *DkMYB20* during ‘Eshi 1’ persimmon fruit development by qRT-PCR. Error bars indicate SEs from three biological replicates (*n* = 3). **(E)** Phylogenetic tree of DkMYB19 and DkMYB20 homologs constructed with the neighbor-joining algorithm using MEGA5. Branches are labeled with the gene names from different plant species. Bootstrap values were calculated from 3000 bootstrap replicates. AtMyb111 (NM_124310.3, *Arabidopsis thaliana*), SlMYB12 (NM_001247472.1, *Solanum lycopersicum*), VvMYBF1(FJ948477.2, *Vitis vinifera*), DkMYB17 (NH210717.1, *Diospyros kaki*), VvMYBPA1 (AM259485.1, *Vitis vinifera*), DkMYB4 (AB503701.1, *Diospyros kaki*), VvMYB5b (AY899404.1, *Vitis vinifera*), OsMYB4 (D88620.1, *Oryza sativa*), DkMYB2 (AB503699.1, *Diospyros kaki*), VvMybPA2 (LOC100264512, *Vitis vinifera*), PtMYB134 (FJ573151.1, *Populus tremuloides*), GhMYB10 (AF336282.1, *Gossypium hirsutum*), LjTT2a (AB300033.2, *Lotus japonicus*), LjTT2b (AB300034.2, *Lotus japonicus*), LjTT2c (AB300035.1, *Lotus japonicus*), SlAnt1 (NM_001247488.1, *Solanum lycopersicum*), InMYB1(AB444398.1, *Ipomoea nil*), LhMYB6 (AB534587.1, *Lilium hybrid*), MtLAP1 (FJ199998.1, *Medicago truncatula*), MdMYB10a (DQ267898.1, *Malus domestica*), MrMYB1 (GQ340767.2, *Morella rubra*), VvMYBA2 (AB097924.1, *Vitis vinifera*), VvMYBA1 (AB097923.1, *Vitis vinifera*), and LeANT1 (AB181203.1, *Lycopersicon esculentum*). **(F)** Subcellular localization of the DkMYB19 and DkMYB20 proteins. The DkMYB19-GFP or DkMYB20-GFP fusion vector or the GFP only vector was transiently expressed into tobacco epidermal cells driven by the CaMV35S promoter and observed under a confocal microscope. Bright-field photographs were taken to examine cell morphology, dark-field images were used to assess for green fluorescence, and the two types of images were combined. The red arrow indicates the nucleus. **(G)** Secondary structure of the miR858b precursor predicted by Mfold. The red box represents the sequence of the miRNA/miRNA* duplex. **(H)** Sequence comparison between the target genes *DkMYB19* and *DkMYB20* and their corresponding miRNA, miR858b. The arrows indicate the cleavage sites, and the numbers show the frequency of correct sequencing clones. **(I)** Co-transformation of *DkMYB19* or *DkMYB20* and miR858b in tobacco leaves. Recombinant vectors were transformed into tobacco leaves by using *Agrobacterium* strain GV3101. The dual-LUC assay showed the interaction of miR858b and its target genes. Relative LUC activity was normalized to Renilla (REN) luciferase activity. Error bars indicate SEs from four biological replicates (*n* = 4). Asterisks above the bars indicate values determined by Student’s *t*-test to be significantly different from the control (*P < 0.05).

Chinese PCNA persimmon genotype ‘Eshi 1’ (*D. kaki* Thunb.; 2n = 6x = 90) was planted in the Persimmon Repository of Huazhong Agricultural University, Wuhan, China. Fruit flesh samples were collected from uniform fruits that were free of visible defects at 2.5, 5, 10, 15, 20, and 25 weeks after blooming (WAB) (Figure 1A). Three biological replicates were collected from three individuals, and each treatment contained 10–15 fruits. All samples were immediately frozen in liquid nitrogen and then stored at −80°C after sampling. Tissue culture seedlings of ‘Gongcheng Shuishi’ (PCA genotype) were grown on MS (1/2N) media in a growth chamber with a 12-h photoperiod and a 24°C temperature. The three positive transgenic seedlings of *DkMYB19* (DkMYB19-22, DkMYB19-37, and DkMYB19-44) and *DkMYB20* (DkMYB20-3, DkMYB20-4, and DkMYB20-9) were propagated for further analysis. Tobacco (*Nicotiana benthamiana*) plants were grown under a 16-h light/8-h dark cycle at 21°C.

### Proanthocyanidin Content Determination

Both soluble and insoluble PA contents were measured by the Folin–Ciocalteau method (Oshida et al., 1996). The PA content was also examined by staining tissues with DMACA (p-dimethylaminocinnamaldehyde) solution (0.6% DMACA and 1% 6 mol/L HCl in methanol) (Li et al., 1996). The infiltrated fruit discs were decolorized in 30% acetic acid in ethanol for 12–20 h. Next, the fruit discs were washed with 75% ethanol and then stained blue in DMACA solution for 2 min. A deeper blue color indicates a higher PA content. The soluble PA content of the fruit during the developed stages was also roughly examined by the printing method according to the previous report by Eaks (1967). The soluble tannin reacts with FeCl_2_, the darker the result is, the higher the soluble tannin content is.

### RNA Extraction and cDNA Synthesis

Total RNA was isolated from fruit flesh using RNAiso Plus^∗^ (Code No.: 9108). The RNA quality and quantity were assessed by gel electrophoresis and Nanodrop 2000 spectrophotometry (Thermo Fisher, United States). Three biological replicates were performed for each sample. For gene isolation, first-strand cDNA was generated using a 2.0-μg total RNA sample with the PrimeScript^TM^ RT reagent kit with gDNA Eraser (Takara, Beijing, China) according to the manufacturer’s protocol. For gene expression analysis, cDNA was synthesized from 1.0 μg of each RNA sample using the PrimeScript RT Kit with gDNA Eraser (TaKaRa, Dalian, China). The reverse transcription of miRNA utilized the Mir-X^TM^ miRNA first-strand synthesis and TB Green^®^ qRT-PCR Kit (TaKaRa, Dalian, China).

### Prediction of Target Genes and miRNA Precursor Secondary Structure

Target gene prediction was performed with the psRNATarget program^1^ based on miRNA (accession number: SRP050516) (Luo et al., 2015) and transcriptome databases (Chen et al., 2017). The main principle of this analysis was to focus on the degree of sequence similarity between the miRNAs and targets. RNA Folding Form software was used to construct the miRNA precursor secondary structure (Zuker, 2003).

### Isolation and Cloning of the *DkMYB19* and *DkMYB20* Genes

In the transcriptome database of C-PCNA persimmon established in our laboratory (Chen et al., 2017), two unigenes (CL4549.Contig2_All and Unigene36868_All) were predicted and screened out as candidate target genes of miR858b, and the full-length sequences of the *DkMYB19* and *DkMYB20* genes were isolated by both 5′/3′ RACE (rapid amplification of cDNA ends) utilizing the SMARTer^®^ RACE 5′/3′ kit based on the original unigene sequences. The primer sequences used for RACE and cloning are described in Supplementary Table S1.

### Sequence Alignment and Phylogenetic Analysis

The gene sequences were translated with online software^2^. The deduced amino acid sequences of the homologous genes of 24 other species were retrieved from the National Center for Biotechnology Information (NCBI). A phylogenetic tree was constructed using the neighbor-joining (NJ) method with MEGA5 software (Tamura et al., 2011).

### Quantitative Reverse Transcription PCR (qRT-PCR)

The quantitative reverse transcription PCR (qRT-PCR) was performed with QuantStudio 7 Flex Real-Time PCR system (Applied Biosystems) using SYBR^®^ Premix Ex TaqTM II (TaKaRa). The PCR mixture (10 μL total volume) included 5 μL of SYBR Premix Ex Taq II (TaKaRa, Dalian, China), 3.5 μL ddH_2_O, 1.0 μL diluted cDNA, and 0.25 μL of each primer (0.01 M). The PCR conditions were as follows: 5 min at 95°C; 45 cycles of 95°C for 5 s, 58°C for 10 s, and 72°C for 15 s. Each sample was assayed in quadruplicate, and *DkActin* (accession no. AB473616) was used as the internal control (Supplementary Table S1). For miRNA, the forward primer was designed based on the sequence, the reverse primer was the universal primer, and the U6 gene was used as the internal control (Supplementary Table S1). All treatments were performed with at least three biological replicates.

### RNA Ligase-Mediated 5′ RACE

RNA ligase-mediated rapid amplification of 5′ cDNA ends (RLM-5′ RACE) was conducted with a GeneRacer kit (Invitrogen, Carlsbad, CA, United States) to map the cleavage sites of the target transcripts. Three replicates of l μg of total RNA isolated from pooled samples of persimmon fruits were subjected to the ligation of 5′ RACE RNA adaptors at 15°C overnight. Gene-specific primers (Supplementary Table S1) were designed to conduct 5′ RACE PCR. The PCR products were cloned into the pEASY-Blunt Simple vector (TransGen Biotech, China) for sequencing.

### Dual-Luciferase Assay Analysis

To verify whether *DkMYB19* and *DkMYB20* could be cleaved by pre-miR858b, they were co-transformed into *N. benthamiana* leaves using an *Agrobacterium*-mediated (GV3101) transfection system. The vector pGreenII0800-Luc, which contains the reporter gene LUC, was used as the expression vector. The ORF sequences of *DkMYB19* and *DkMYB20* without the termination codon were fused to pGreenII800-Luc after endonucleases digestion with *Kpn* I/*Bam*H I; the primers are listed in Supplementary Table S1.

To further verify the interaction between *DkMYB20* and *DkANR* promoter, the *DkANR* promoter was fused to pGreenII0800-LUC to act as reporter, the ORF sequence of *DkMYB20* was fused to pGreenII 62-SK to act as effector, the primers are listed in Supplementary Table S1. The plasmids were transformed into *Agrobacterium* GV3101 after sequence verification. Transient expression in tobacco leaves was performed according to Lee et al. (2008) and Li (2011). The activity detection of LUC (Firefly Luciferase) and REN (Renilla Luciferase) were performed with the Dual-Luciferase^®^ Reporter Assay System (Promega, United States) kit.

### Subcellular Localization of DkMYB19 and DkMYB20

The complete open reading frames (ORFs) of *DkMYB19* and *DkMYB20* without the termination codon were amplified using the corresponding primers (Supplementary Table S1) to construct the fusion constructs 35S:DkMYB19-GFP and 35S:DkMYB20-GFP according to Guan et al. (2016), and the 35S-GFP vector served as a positive control. The fusion and control plasmids were transferred into *Agrobacterium tumefaciens* strain GV3101 via heat shock and then transiently transformed into the leaves of 6-week-old *N. benthamiana* plants. Three days later, the fluorescence signals were detected under a fluorescence microscope (Nikon 90i, Japan) (Zhao et al., 2019).

### Verification of the Targeting of *DkMYB19*/*DkMYB20* by miR858b Through the GFP Signal Detection

To verify that the *DkMYB19*/*DkMYB20* was harbored by miR858b, we constructed five vectors (Supplementary Table S1) and performed a transient expression in tobacco leaves *in vivo* with the pMS4 vector. The pre-miR858b sequence was ligated to the pMS4 vector with the *Xho* I/*Xba* I directly, the 26-bp length that contained the target site or mutated target site (CCCCCC) in the sequence of *DkMYB19*/*DkMYB20* were synthesized by primers self-linking. The reaction system for primer self-linking was as follows: 10 × T4 DNA Ligase Buffer 4 μL, primer-F 18 μL, and primer-R 18 μL; the mix solution was incubated at 55°C for 20 min, then the products were ligated into pMS4 vectors with the *Xho* I/*Xba* I. Transient expression in tobacco leaves *in vivo* was used as described by Li (2011). Three days later after infiltration, the GFP signal was visualized under bright light and UV light, respectively.

### Transient Expression in Persimmon Leaves *in vivo*

A transient overexpression system was utilized to assess the role of miR858b, *DkMYB19*, and *DkMYB20* in regulating PA biosynthesis in persimmon leaves *in vivo*. The full-length pre-miR858b, *DkMYB19*, and *DkMYB20* gene sequences were inserted into the plant expression vector pMDC32 by homologous recombination according to Mo et al. (2016), while the GFP-containing pMDC32 vector was used as the control; the primers are listed in Supplementary Table S1. The short tandem target mimic (STTM) of the miR858b (STTM858b) module was inserted into the pMDC32 vector between the 2 × 35S promoter and the 35S terminator using a reverse PCR procedure according to Tang et al. (2012) with the primers described in Supplementary Table S1. The construct was transferred into persimmon leaves via a previously described *Agrobacterium*-mediated method, with minor modifications (Mo et al., 2015) as follows: The *Agrobacterium* cells were collected after centrifugation and then resuspended to obtain an optical density (OD) at 600 nm of 0.75. The transformed leaves continued to grow on the tree until they were sampled for qRT-PCR and PA content analysis. Ten days after the injection of these constructs into leaves, 100 mg of tissue from each infiltrated leaf was collected for further analysis, each treatment was performed with three biological replicates, and each replicate treatment included 10 leaves.

### Transient Transformation in Persimmon Fruit Discs *in vitro*

We introduced the transient transformation system into persimmon fruit discs. Discs of 1 cm in diameter and 0.2 cm in thickness were incubated for 1 h in *Agrobacterium* infection solution carrying the constructs. The discs were then transferred to filter paper wetted with MS liquid medium in tissue-culture dishes and placed in an incubator at 25°C for 3 days. All of the treatments involving all genes and the empty vector were performed with three biological replicates. At each sampling point, the discs were dried on filter paper, frozen in liquid nitrogen, and then stored at −80°C for further analysis (Zhu et al., 2018).

### Transformation of Wild-Type *Arabidopsis*

The *A. tumefaciens* GV3101 line containing 2 × 35S:pre-miR858b was introduced into wild-type (WT) *Arabidopsis thaliana* according to the *in planta* transformation procedure of Zhang et al. (2006). The seeds of the WT and the 2 × 35S:pre-miR858b lines were germinated on MS medium containing 6% sucrose and 50 mg/L kanamycin, followed by transplantation into a culture substrate to generate T1 seeds.

### Production of Transgenic Persimmon Leaf Calli

The transformation of persimmon was performed with *A. tumefaciens* according to the methods reported by Tao et al. (1997). Discs of leaves were infected with transformed *A. tumefaciens*, and callus tissue was regenerated on solid MS(1/2N) medium containing 50 mg/L kanamycin as a selective antibiotic.

### Yeast One-Hybrid Assays

The full-length CDS of *DkMYB20* was inserted into the pGADT7 vector, and the promoter of *DkANR* was subcloned into the pAbAi vector. Y1H was performed to confirm the interactions between DkMYB20 and *DkANR* using the Matchmaker^TM^ Gold Yeast One-Hybrid Library Screening System (Clontech, United States). Both fusion constructs were transformed into the Y1H Gold strain and cultured on SD/-Urp medium containing 100 ng/mL aureobasidin A (AbA). pGADT7 (AD-P53) and p53AbAi were used as positive controls.

### Yeast Two-Hybrid Assays

To detect proteins that may interact with *DkMYB19* and *DkMYB20* in persimmon, yeast two-hybrid (Y2H) assays were performed. The full-length coding sequences of *DkMYB19* and *DkMYB20* were amplified by PCR using cDNA as the template (Supplementary Table S1) and then ligated into the pGBKT7 [binding domain (BD)] bait vector via *Bam*H I and *Sma* I double digestion. For Y2H screening, an expressed cDNA library from C-PCNA ‘Luotian Tianshi’ fruit was inserted into the pADGAL4 prey vector via recombination. The resulting bait and prey vectors were transformed into the yeast strain AH109 by the LiAc/DNA/PEG method according to the Matchmaker^®^ Gold Y2H System user manual. Co-transformed yeast cells were first plated on SD medium lacking leucine and tryptophan (SD–Leu–Trp) and incubated at 30°C for 3–5 days. Transformed yeast cells containing the pGBKT7-53 + pGADT7-T, pGBKT7-Lam + pGADT7-T, and pGBKT7 constructs were used as positive, negative, and blank controls, respectively. pGBKT7-*DkMYB19* and pGBKT7-*DkMYB20* were transformed into yeast cells in self-activation activity assays. Single colonies were patched on quadruple selection SD medium lacking adenine, histidine, leucine, and tryptophan (SD–Ade–His–Leu–Trp), followed by incubation at 30°C for 3–5 days. Yeast colony PCR using 5′ and 3′ PCR primers (Supplementary Table S1) was performed to determine the presence of inserts in the prey pGADT7-Rec clones.

Plasmids were isolated from yeast colonies derived from the QDO selective media using the Dr GenTLE^®^ (from Yeast) High Recovery, and the “prey” vectors containing inserts of candidate interactors were isolated by transformation into Trans1-Blue Chemically Competent Cells, which were then plated on LB with ampicillin (Amp) (selective for only pGADT7-Rec clones). Colonies were picked and cultured in LB/Amp (overnight), and the plasmids were purified. The protein–protein interactions (PPIs) were confirmed by co-transforming Y2H gold cells with the bait (*DkMYB19* in pGBKT7) clone together with the interactor prey clone (in pGADT7-Rec) and plating them on QDO. To check for any false-positive interactions, the empty bait vector was co-transformed with the interactor prey clone, followed by plating as indicated above. The pGADT7-Rec clones were sequenced in the forward and reverse directions using T7 and 3′AD primers (Eurofins, India). The sequences of the identified interacting proteins were subjected to BLASTN and BLASTX (NCBI^3^) analyses to identify and confirm the correct orientation of the interactor sequences and to rule out any false-positive results or large ORFs in the incorrect reading frame (Ramalingam et al., 2015).

## Results

### PA Accumulation Continued Until the Final Stage of Fruit Development in C-PCNA

The imprinting method was used to determine the PA contents of persimmon fruits. The sections were deeply stained at the beginning of fruit development (2.5 WAB), when the young fruits of ‘Eshi 1’ were small in size. With the progression of development, the fruits grew quickly and became increasingly larger until reaching their largest sizes at 25 WAB (Figure 1A). The fruit sections were still stained dark by FeCl_2_ up to 15 WAB, but the staining began to diffuse at 20 WAB. In the last developmental stage of 25 WAB, the fruits were only lightly stained (Figure 1B). To confirm the imprinting results, quantitative measurements of soluble and insoluble PA contents in the fruits were carried out using the Folin–Ciocalteau method. The soluble PA content in the fruits was 2.098 mg/g at 2.5 WAB but quickly decreased until 15 WAB (0.502 mg/g), with only a slight increase being observed at 20 WAB, followed by a decrease to the lowest level (0.245 mg/g) at 25 WAB, which indicated that the fruits had already lost their astringency and tasted without astringent sensation (drying or puckering feeling) in the mouth. Insoluble PA, which exhibited markedly lower levels than soluble PA, showed a trend of continuous decline except for a transient increase from 10 to 15 WAB; it reached its peak level (0.712 mg/g) at 15 WAB and lowest level (0.473 mg/g) at 25 WAB (Figure 1C).

### MiR858b and *DkMYB19* or *DkMYB20* Showed Divergent Expression Patterns During Fruit Development

The relative expression of miR858b was very low before 20 WAB and then sharply increased to the maximum level at 20 WAB, followed by a slight increase at 25 WAB. The transcript level of *DkMYB19* presented upregulation at 5 WAB, after which it tended to decrease from 5 to 25 WAB; in contrast, the level of *DkMYB20* decreased approximately 70% at 5 WAB, followed by a marked upregulation at 10 WAB, after which it showed a trend of downregulation from 10 to 20 WAB, and then slight upregulation at 25 WAB (Figure 1D).

### Isolation and Sequence Analyses of *DkMYB19* and *DkMYB20* in C-PCNA Persimmon

*DkMYB19*, consisting of 633 base pairs, was isolated and predicted to encode a protein of 210 amino acid residues, while *DkMYB20* consisted of 771 base pairs and was predicted to encode a protein of 256 amino acid residues. Both proteins contained an N-terminal R2R3-DNA BD (Supplementary Figure S2). *DkMYB19* and *DkMYB20* were both clustered into the PA-related MYB clade. *DkMYB19* was closely related to *DkMYB17* (MH210717.1, *D. kaki*); the corresponding protein sequences showed 95% similarity. *DkMYB20* was phylogenetically closer to *DkMYB2* (AB503699.1, *D. kaki*), which has been previously described as a PA biosynthesis regulator (Akagi et al., 2010), and the protein sequence similarity was 98% (Figure 1E).

### DkMYB19 Localized to the Nucleus as Well as Cytoplasm and DkMYB20 Localized to the Nucleus

Most R2R3 MYB genes are exclusively localized and function in the nucleus (Stracke et al., 2001). Similar to other R2R3 MYB TFs, the MYB19-GFP is present throughout the cytoplasm as well as the nucleus. MYB20-GFP is located predominantly in the nucleus, whereas GFP (control) was uniformly distributed throughout the cell (Figure 1F).

### Validation of the Target Genes of miR858b

In the Mfold output, persimmon-specific miRNA and miRNA^∗^ were incorporated in the backbone, and the guiding strands of the miRNAs were considered without changing any other nucleotide (Figure 1G).

The sequencing of the 5′ RACE PCR clones demonstrated that pre-miR858b exhibited unique cleavage sites in the corresponding target sequences at 10–11 nt sites (Figure 1H). LUC/REN (Renilla) in the leaves inoculated with GV3101-pGreenII 0800-Luc (control) showed the same result as that in the leaves inoculated with GV3101-pGreenII 0800-Target-Luc, in which the target sequence was fused upstream of the LUC gene. LUC/REN activity decreased in leaves co-transformed with GV3101-pGreenII0800-Target-Luc and GV3101-pGreen62-SK-pre-miR858b. In agreement with the RLM-5′ RACE sequencing results, miR858b could cleave the candidate targets (Figure 1I).

**FIGURE 2:**
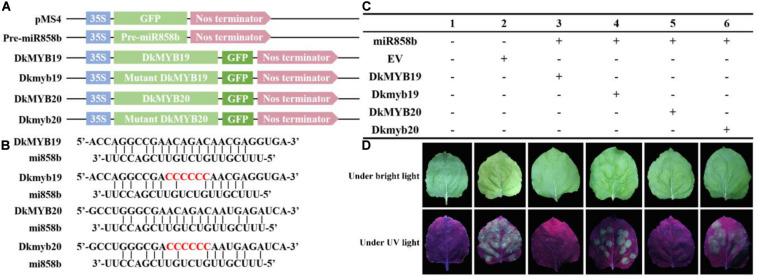
The co-expression of *MIR858b* and its target genes by *Agrobacterium tumefaciens*-mediated transformation in tobacco leaves. **(A)** Six vectors designed for transient expression. **(B)** The DkMYB19/DkMYB20 were the vectors carrying the target sites of miR858b, the Dkmyb19/Dkmyb20 were the vectors carrying the mutated target sites. The red characters represented the modified sequence at the target sites. **(C)** Different vectors combine used in tobacco co-expression assays. **(D)** The GFP signal was detected under bright light and UV light (λ = 356 nm) at 3 days after infiltration.

In addition, the GFP signal in the tobacco leaves infiltrated with miR858b and DkMYB19 or DkMYB20 almost could not be detected, but it was strong in the tobacco leaves infiltrated with miR858b and Dkmyb19 or Dkmyb20; similar results were obtained in the leaves infiltrated with pMS4 (Figure 2). This result indicated that the miR858b repressed the expression of *DkMYB19*/*DkMYB20* by targeting it at the target site (ACAGAC).

### Transient Expression in Persimmon Leaves *in vivo*

**FIGURE 3:**
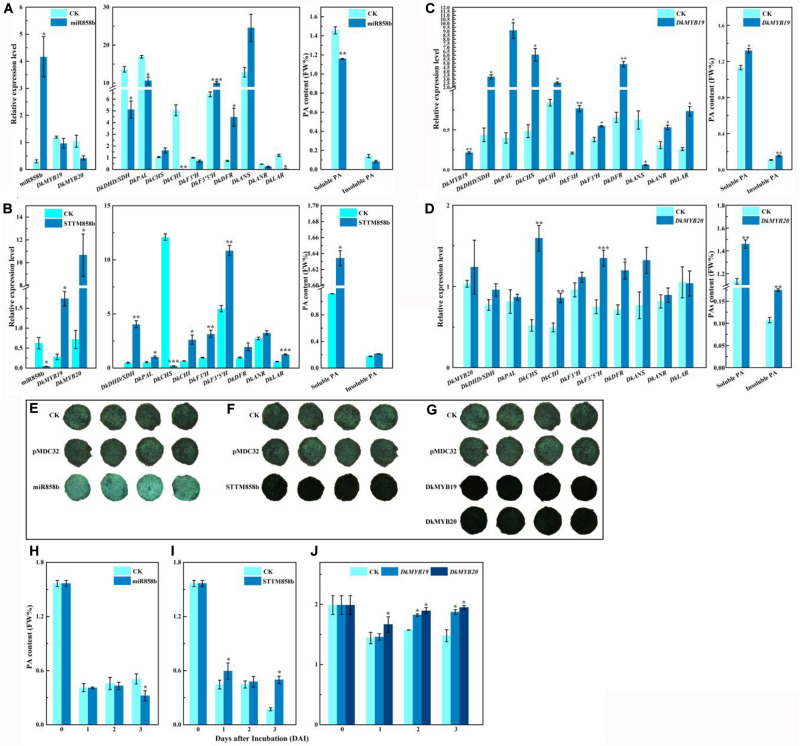
Transient expression of miR858b, *DkMYB19*, and *DkMYB20* in ‘Eshi 1’ persimmon leaves *in vivo* or fruit discs *in vitro*. **(A)** Analysis of the transcript levels of miR858b, *DkMYB19*, *DkMYB20*, and PA biosynthesis pathway genes and the corresponding variation in PA contents after the transient overexpression of pre-miR858 at 10 days after agroinfiltration. **(B)** Analysis of the transcript levels of miR858b, *DkMYB19*, *DkMYB20*, and PA biosynthesis pathway genes and the corresponding variation in PA contents after the transient expression of STTM858b at 10 days after agroinfiltration. **(C)** Analysis of the transcript levels of *DkMYB19* and PA pathway genes and the corresponding variation in PA contents after the transient expression of *DkMYB19* at 10 days after agroinfiltration. **(D)** Analysis of the transcript levels of *DkMYB20* and PA pathway genes and the corresponding variation in PA contents after the transient expression of *DkMYB20* at 10 days after agroinfiltration. Errors bars indicate SEs from three biological replicates (*n* = 3). The PA content variation in ‘Eshi 1’ persimmon fruit discs transformed with miR858b **(E,H)**, *DkMYB19* **(F,I)**, and *DkMYB20* **(G,J)**, assessed by DMACA staining **(E–G)** and the Folin–Ciocalteau **(H–J)** method. The DMACA staining of fruit discs of ‘Eshi 1’ was performed 3 days after infection. CK, blank control; pMDC32, empty vector. Errors bars indicate SEs from three biological replicates (*n* = 3). Asterisks above the bars indicate values determined by Student’s *t*-test to be significantly different from the control (*P < 0.05, **P < 0.01, ***P < 0.001).

The matching expressions between miR858b and *DkMYB19* or *DkMYB20* indicated that miR858b is a suppressor of expressions of *DkMYB19* and *DkMYB20*. As shown in Figure 3A, miR858b was sharply upregulated by 13.805-fold in the leaves infiltrated with pre-miR858b relative to the control, the transcript abundances for *DkMYB19* and *DkMYB20* were lower in the pre-miR858b infiltrated leaves than that in the control, but not to a statistically significant extent, which led to a significant downregulation of *DkDHD*/*SDH* (3-dehydroquinate dehydratase/shikimate 5-dehydrogenase), *DkPAL* (phenylalanine ammonia-lyase), *DkCHI* (chalcone isomerase), and *DkLAR*. As expected, the contents of soluble PA decreased significantly; meanwhile, insoluble PA was lower in the pre-miR858b infiltrated leaves, though it did not reach to a statistically significant extent (Figure 3A). Moreover, a miR858b blocking construct, STTM858b, was generated and infiltrated into leaves *in vivo*. We found that miR858b was sharply downregulated by 93.357% compared to the control, while *DkMYB19* and *DkMYB20* were upregulated significantly, together with the significant upregulation of *DkDHD/SDH*, *DkPAL*, *DkCHI*, *DkF3′H*, *DkF3′5′H*, and *DkLAR*, and the transcript abundances for *DkDFR* (dihydroflavonol 4-reductase) and *DkANR* were higher in the STTM858b infiltrated leaves without a statistically significant extent. As a consequence, the soluble PA content increased significantly but not for the insoluble PA content in the STTM858b infiltrated leaves (Figure 3B).

In the *DkMYB19*-infiltrated leaves, the expression level of *DkMYB19* increased fourfold compared with the control (Figure 3C), while the transcript abundance for *DkMYB20* was higher in the *DkMYB20* infiltrated leaves with no statistically significant extent (Figure 3D). QRT-PCR analysis revealed that the transient expression of *DkMYB19* induced the significant upregulation of *DkDHD/SDH*, *DkPAL*, *DkCHS*, *DkCHI*, *DkF3H* (flavanone3-hydroxylase), *DkF3′H*, *DkDFR*, *DkANR*, and *DkLAR*, and *DkMYB20* induced the significant upregulation of *DkCHS*, *DkCHI*, *DkF3′5′H*, and *DkDFR*, while the expressions of *DkDHD/SDH*, *DkPAL*, *DkF3′H*, *DkANS*, and *DkANR* were higher but not statistically significant in the *DkMYB20-*infiltrated leaves than the control. All infiltration experiments stimulated increases in the soluble PA contents and insoluble PA significantly (Figures 3C,D).

### Transient Transformation in Fruit Discs *in vitro*

When pre-miR858b was infiltrated into fruit discs, DMACA staining was distinctly lighter than in the control (Figure 3E), which meant that the PA content had decreased (Figure 3H). After STTM858b was infiltrated in fruit discs, the DMACA staining of fruit discs was distinctly darker compared with the control (Figure 3F), which implied that the PA content had increased (Figure 3I). DMACA staining was distinctly darker in the fruit discs infiltrated with either *DkMYB19* or *DkMYB20* compared with the control (Figure 3G). The PA content increased accordingly (Figure 3J). Thus, we concluded that miR858b repressed the expression of *DkMYB19* and *DkMYB20*, which are involved in the regulation of persimmon PA biosynthesis.

### Genetic Transformation in Wild-Type *Arabidopsis* and Persimmon Leaf Calli

**FIGURE 4:**
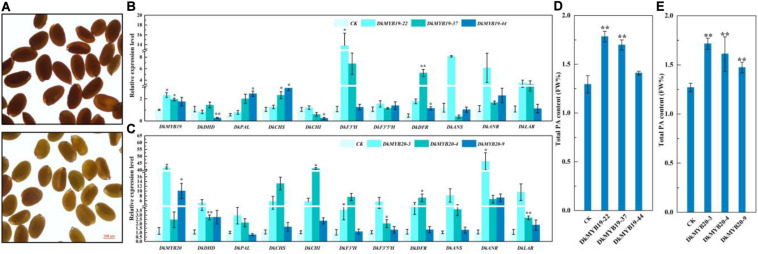
Functional analysis of miR858b, *DkMYB19*, and *DkMYB20* with a stable transformation system. **(A)** Images of wild-type (WT) *Arabidopsis* seeds (upper) and T_1_ *Arabidopsis* transgenic lines (lower) derived from 2 × 35S:pre-miR858 transformation in the WT background are presented. Scale bar = 200 μm. **(B,D)** Analysis of the transcript levels of PA biosynthesis pathway genes and PA content variation in the *DkMYB19* transgenic ‘Gongcheng Shuishi’ plants. **(C,E)** Analysis of the transcript levels of PA biosynthesis pathway genes and the corresponding variation of PA contents in the *DkMYB20* transgenic ‘Gongcheng Shuishi’ plants. Errors bars indicate SEs from three biological replicates (*n* = 3). Asterisks above the bars indicate values determined by Student’s *t*-test to be significantly different from the control (*P < 0.05, **P < 0.01).

MiR858b is a conserved miRNA found in *A. thaliana*, and we transformed 2 × 35S:pre-miR858b into WT *Arabidopsis*. The T_1_ seed coats of the transgenic lines were paler than their brown WT counterparts (Figure 4A). Through stable genetic transformation in leaf calli of ‘Gongcheng Shuishi,’ we obtained five positive transgenic seedlings for the *DkMYB19* gene and 15 transgenic seedlings positive for the *DkMYB20* gene. Three regenerated positive seedlings transformed with CaMV35S-sense *DkMYB19*, referred to as the *DkMYB19-22*, *DkMYB19-37*, and *DkMYB19-44* lines, in which the expression level of *DkMYB19* was upregulated 1.34-fold, 0.95-fold, and 0.75-fold, respectively (Figure 4B), and showed high accumulation of PA (Figure 4D). The qRT-PCR analysis of these three *DkMYB19* transgenic lines revealed that the upregulation of *DkMYB19* was accompanied by proportional increases in the expression levels of some structural genes of the PA biosynthesis pathway, including *DkPAL*, *DkCHS*, *DkF3′H*, *DkDFR*, *DkANR*, and *DkLAR* (Figure 4B). The three *DkMYB20*-positive seedlings, designated as the *DkMYB20-3*, *DkMYB20-4*, and *DkMYB20-9* lines, in which the expression level of *DkMYB20* was upregulated 61.13-fold, 6.74-fold, and 13.80-fold, respectively (Figure 4C), also showed the accumulation of PA (Figure 4E). The expression levels in the three *DkMYB20* lines revealed that the upregulation of *DkMYB20* promoted the expression of the following PA biosynthesis pathway structural genes: *DkDHD/SDH*, *DkCHS*, *DkCHI*, *DkF3′H*, *DkF3′5′H*, *DkDFR*, *DkANS*, *DkANR*, and *DkLAR* (Figure 4C). All of these results indicated that *DkMYB19* and *DkMYB20* directly acted as regulators of PA pathway genes and controlled PA biosynthesis in persimmon.

### DkMYB20 Activated the *DkANR* Promoter

**FIGURE 5:**
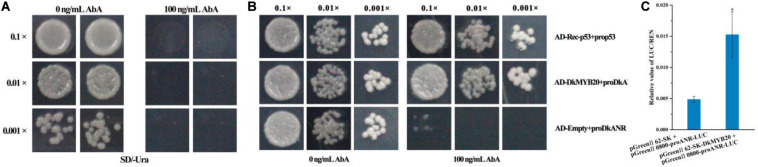
DkMYB20 activated the *DkANR* promoter. **(A)** Screening of the minimal inhibitory concentration of AbA for the *DkANR* bait yeast strain. **(B)** Yeast one-hybrid assays. AD-Rec-p53 with prop53 was used as the positive control, and AD-empty with proDkANR was used as the negative control. Asterisks above the bars indicate values determined by Student’s *t*-test to be significantly different from the control. **(C)** Interaction between *DkMYB20* and *DkANR* promoter through LUC analysis (*P < 0.05).

Linearized *DkANR*-AbAi was transformed into Y1H Gold cell, which were then grown on SD/-Ura medium with aureobasidin A (AbA), and we found that the *DkANR* promoter was suppressed by 100 ng ml^–1^ AbA (Figure 5A). In the interaction tests, the expression of *DkMYB20* induced the expression of the AbA-resistance reporter gene driven by the *DkANR* promoter (Figure 5B). In dual-luciferase assay, the relative value of LUC/REN in the leaves that co-transformed pGreenII 62-SK-DkMYB20 and pGreen II 0800-proANR-LUC was 3.136-fold compared to that co-transformed pGreen II 62-SK and pGreen II 0800-proANR-LUC, which also indicated that *DkMYB20* activated the *DkANR* promoter (Figure 5C).

### The Interaction Between DkMYB19 and DkPK2

We found that DkMYB19 could interact with the DkPK2 protein, which is a pyruvate kinase that may be involved in the natural loss of astringency in C-PCNA persimmon mediated by the upregulation of *DkPDC* (pyruvate decarboxylase) and alcohol dehydrogenase (*DkADH*) expression during the last developmental stage (Guan et al., 2016, 2017). The pGBDT7–DkMYB19 and pGADT7–DkPK2 fusion proteins were co-transformed, and the Y2H assay verified the interactions (Supplementary Figure S3).

## Discussion

Persimmon is an ideal material for studying PA metabolism because it can specifically accumulate PA in its tannin cells. Two MYB-TFs (*DkMYB2* and *DkMYB4*) are key regulators involved in PA biosynthesis in persimmon (Akagi et al., 2009, 2010). *DkMYB4* is a predominant regulator controlling PA biosynthesis in Japanese PCNA persimmon, and the downregulation of *DkMYB4* in the early stages of fruit development leads to a substantial downregulation of PA pathway genes, which results in the loss of astringency. *DkMYB2* can directly bind to the AC-rich *cis*-motifs known as AC elements in the promoters of *DkANR* and *DkLAR*, which control PA metabolism in persimmon (Akagi et al., 2010). Sequence analysis showed that *DkMYB20* shares 93% identity with *DkMYB2* and that *DkMYB19* shares 94% identity with *DkMYB17* (NCBI Accession No.MH210717.1), which may be involved in persimmon fruit postharvest deastringency (data unpublished). Our data presented here indicate a novel mechanism integrating translational regulation to control *DkMYB19* and *DkMYB20* expression in the PA biosynthesis pathway in persimmon.

A major group of miRNA targets is composed of the relatively unknown MYB genes. MiR858 can target 36% of MYBs, and miR828 targets 15%, with a TAPIR target prediction score of 6. MiR858 was initially functionally identified in ***Arabidopsis*** (Rajagopalan et al., 2006) and more recently in apple (***Malus domestica***) (Xia et al., 2012) and cotton (***Gossypium hirsutum***) (Guan et al., 2014). In apple, miR858 targets up to 66 MYB factors (Xia et al., 2012). As miR858 may be involved in numerous biological processes and metabolic pathways, its diverse functions remain to be elucidated. Recently, miR858 has been shown to regulate ***MYB2*** gene homologs that function in ***Arabidopsis*** trichome and cotton fiber development (Guan et al., 2014) and several R2R3 MYB TF genes, including ***AtMYB12*** (At2g47460), ***AtMYB13*** (At1g06180), ***AtMYB20*** (At1g66230), and ***AtMYB111*** (At5g49330) (Addo-Quaye et al., 2008), among which ***MYB12*** and ***MYB111*** are associated with flavonoid metabolism in ***Arabidopsis*** seeds (Stracke et al., 2007). MiR828 and miR858 have been reported to regulate ***VvMYB114*** to promote anthocyanin and flavonol accumulation in grapes (Tirumalai et al., 2019).

The major targets of miR858b identified in our analysis were two MYBs, *DkMYB19* and *DkMYB20*. The persimmon miR858b sequence, screened for the first time in this study, displayed significant upregulation of expression at 20 and 25 WAB, which caused the downregulation of the two *MYB* TFs. These findings are in agreement with the hypothesis that miR858 might target a number of R2R3-MYB genes involved in the secondary metabolite pathway, and even slight variation in miR858 expression could exert an obvious influence on product accumulation.

Compared with the WT, transient overexpression of miR858b suppressed the expression of *DkMYB19* and *DkMYB20* and key PA pathway genes (Figures 3A,E,H), while transient silencing of the expression of miR858b upregulated the expression of *DkMYB19* and *DkMYB20* and key PA pathway genes (Figures 3B,F,I), the transient overexpression of *DkMYB19* or *DkMYB20* could also upregulate key PA pathway genes (Figures 3C,D,G,J). This result indicated that the repression of *DkMYB19* and *DkMYB20* by miR858b is involved in regulating PA accumulation. In particular, *DkMYB20* specifically regulated the expression of *DkANR*, and the interaction relationship was validated through Y1H assays and LUC/REN measurement. The regulatory activity of MYB TFs in recognizing and binding DNA with a high affinity and specificity has been suggested to involve PPIs and posttranslational modifications (PTMs) (Dubos et al., 2010). In this study, the Y2H approach was used to screen a C-PCNA fruit cDNA library to identify the complete coding sequences (CDSs) encoding PA metabolism proteins as potential interacting partners. Our results suggested that the *DkPK2* gene and PPI may play relevant roles in the regulation of PA biosynthesis.

**FIGURE 6:**
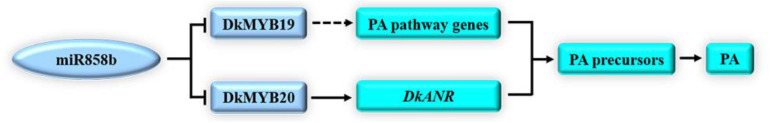
A hypothetical model of the involvement of miR858b, *DkMYB19*, and *DkMYB20* in PA biosynthesis. In the present study, miR858b directly repressed the transcription of *DkMYB19* and *DkMYB20* in the posttranscriptional step. *DkMYB20* contributed to regulating *DkANR* to control PA biosynthesis, and *DkMYB19* may regulate some of the PA pathway genes involved in PA biosynthesis in C-PCNA persimmon.

Based on our data and earlier studies, we proposed a model for the regulation of miR858b in the PA biosynthesis pathway in C-PCNA persimmon (Figure 6). The results directly indicated that miR858b repressed the expression of *DkMYB19* and *DkMYB20*, while *DkMYB20* may contribute to regulating PA pathway genes to control PA biosynthesis, and *DkMYB19* could regulate *DkPK2* to influence the expression of *ADH* and *PDC*, involved in the natural loss of astringency in C-PCNA persimmon. Moreover, the transient transformation of persimmon fruit discs *in vitro* provides credible evidence of the functions of miR858b, *DkMYB19*, and *DkMYB20*.

## Conclusion

We isolated miR858b, which repressed the expression of *DkMYB19* and *DkMYB20* and negatively regulated PA biosynthesis. These results provide new scientific evidence further elucidating the regulatory network of PA metabolism and may contribute to the breeding of novel PCNA cultivars.

## Data Availability Statement

The datasets presented in this study can be found in online repositories. The names of the repository/repositories and accession number(s) can be found in the article/Supplementary Material.

## Author Contributions

SY designed and conducted all experiments, analyzed the data, and wrote the manuscript. MZ conducted a portion of the experiments. LX and ZL provided advice about the experiments and revised the manuscript. QZ conceived the project and designed the experiments. All authors read and approved the manuscript.

## Conflict of Interest

The authors declare that the research was conducted in the absence of any commercial or financial relationships that could be construed as a potential conflict of interest.

## Footnotes

1 http://plantgrn.noble.org/psRNATarget/

2 http://linux1.softberry.com/

3 http://www.ncbi.nlm.nih.gov/
