# Supplementary material for: MiR858b Inhibits Proanthocyanidin Accumulation by the Repression of DkMYB19 and DkMYB20 in Persimmon
Source: Front Plant Sci. 2020 Dec 21;11:576378. doi: 10.3389/fpls.2020.576378 (PMC7779590; doi:10.3389/fpls.2020.576378)
Supplement: Supplementary file 1 [file Data_Sheet_1.PDF]

Supplementary Material

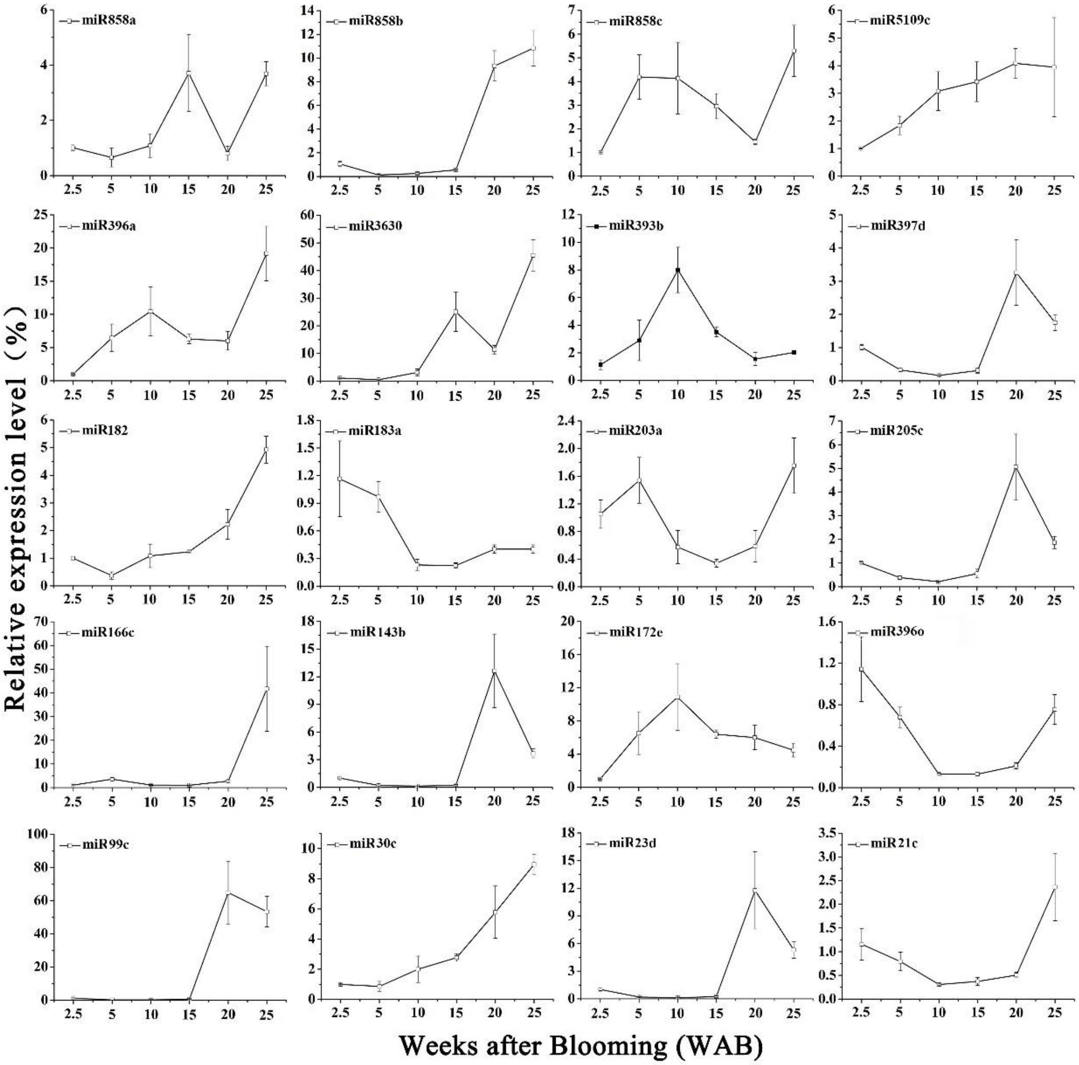

Figure S1. QRT-PCR analysis of conserved miRNA during fruit development in 'Eshi 1' persimmon. Flesh was collected at 2.5, 5, 10, 15, 20, and 25 WAB. WAB, weeks after blooming. Error bars indicate the standard deviation (n = 3).

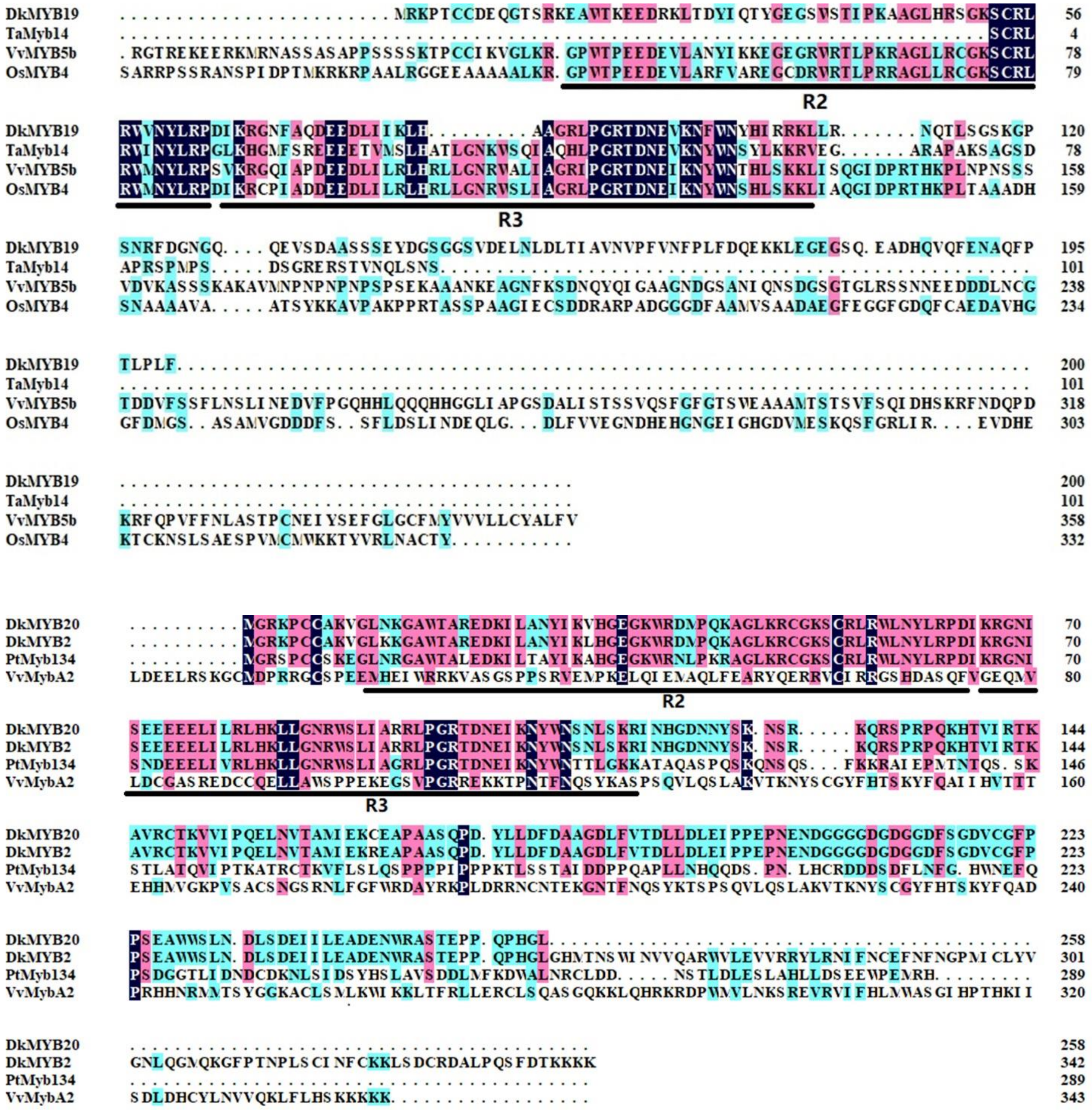

Figure S2. Protein sequence alignment of DkMYB19, DkMYB20 and other R2R3-MYB transcription factors from various plant species. Identical residues marked in black, conserved residues in dark gray, and similar residues in light gray. The lines under the alignment located the different domains and motifs within the proteins. The DNA binding domain corresponds to the R2R3-MYB repeats.

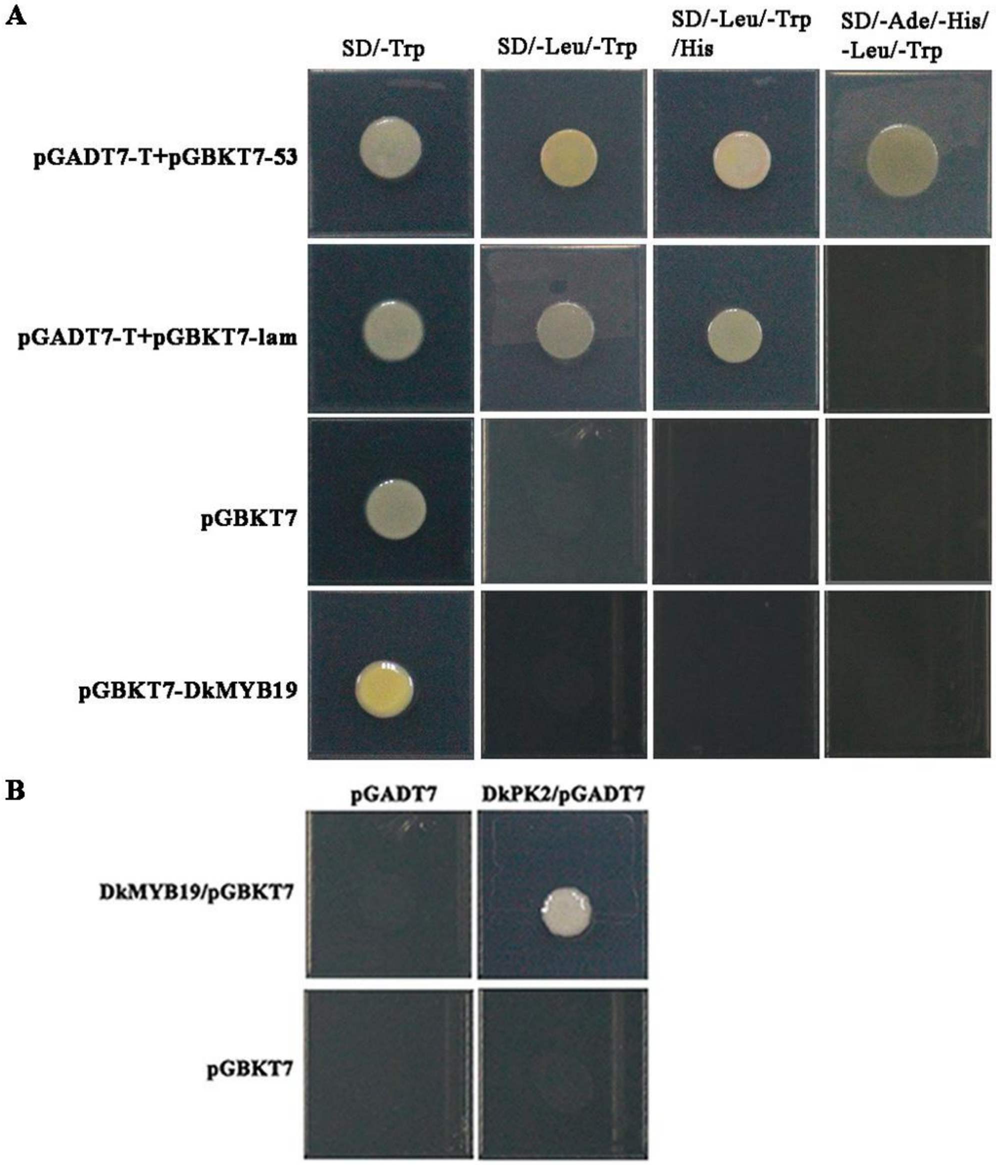

Figure S3. Yeast-two-hybrid assay. (A) Self-activation activity detection. pGADT7-T+pGBKT7-53 were used as positive control; pGADT7-T+pGBKT7-lam, pGADT7-T+pGBKT7-lam were used as negative controls; pGBKT7 were used as blank control; (B) Yeast two-hybrid assays of the interactions between DkMYB19 and DkPK2. The empty vectors of pGBKT7 and pGADT7 were used as negative controls.
